# *Hanguanadeflexa* (Hanguanaceae), a new forest species from Sarawak, Borneo

**DOI:** 10.3897/phytokeys.181.69045

**Published:** 2021-08-18

**Authors:** Michal Hroneš, Jana Leong-Škorničková, Matti A. Niissalo, Martin Dančák

**Affiliations:** 1 Department of Botany, Palacký University, Šlechtitelů 27, Olomouc, CZ-78371, Czech Republic; 2 Singapore Botanic Gardens, National Parks Board Singapore, 1 Cluny Road, 259569, Singapore, Singapore; 3 Department of Ecology and Environmental Sciences, Palacký University, Šlechtitelů 27, Olomouc, CZ-78371, Czech Republic

**Keywords:** Commelinales, Malaysia, rainforest, taxonomy

## Abstract

*Hanguanadeflexa***sp. nov.** (Hanguanaceae) from Lawas district, Sarawak, Malaysia (northern Borneo) is described and illustrated, bringing the total number of species in Borneo to eight. The new species differs from all other recognized *Hanguana* species by a combination of flat leaf blade, deflexed infructescences, one-seeded dull red fruits with centrally positioned stigma and globose seed with wedge-shaped ostiole. Revised key for Bornean *Hanguana* species is presented.

## Introduction

The flora of Borneo is one of the richest in the World (Kier et al. 2005). At the same time, it is among the most threatened, especially due to forest logging, conversion of forest to cropland and large-scale infrastructure projects (Wilcove et al. 2013; Alamgir et al. 2019). Knowledge regarding the taxonomy and diversity of many plant groups is far from perfect and some species probably become extinct before they are even discovered. An illustrative example of such groups are the monocot herbs (so called macroherbs; Siti Nurfazilah et al. 2011). While the diversity of some families is well to moderately well explored (e.g. Araceae and Zingiberaceae; Smith 1985, 1986, 1987, 1988, 1989; Boyce and Julia 2006; Boyce and Wong 2015, 2019, and to some extent also Lowiaceae and Taccaceae; Poulsen and Leong-Škorničková 2017; Syauqina et al. 2019; Wong and Chua 2019; Leong-Škorničková et al. 2021), the diversity of others remains poorly understood (e.g. Hypoxidaceae, Hanguanaceae). The herbarium taxonomy of these groups suffers from the scarcity of well-preserved herbarium specimens documenting the full set of morphological characters (i.e. flowers and fruits) and high superficial similarity of their vegetative parts (e.g. Boyce and Julia 2006; Siti Nurfazilah et al. 2011; Leong-Škorničková and Niissalo 2017). Consequently, the existing flora accounts usually contain very broad species circumscription, e.g. Flora Malesiana accounts for *Curculigo* (Geerinck 1993), *Hanguana* (Backer 1951) and *Tacca* (Drenth 1976).

*Hanguana* Blume, the sole genus of the family Hanguanaceae, is distributed from Sri Lanka and Myanmar to northern Australia (POWO 2019). Currently, 21 species are recognized of which *Hanguanaanthelminthica* (Blume ex Schult. and Schult.f.) Masam. covers the whole distribution range of the genus, while the vast majority of all other species have very narrow ranges (Leong-Škorničková and Niissalo 2017; Randi et al. 2021). The species are dioecious. However, males are only rarely observed and for numerous species, male plants remain unknown. As many female plants produce viable seeds in the absence of males the discovery of polyploidy and apomixis in some species (Niissalo et al. 2020) is not surprising. Consequently, only female individuals in the mature fruiting stage are usually used for description of new taxa. Morphological traits for species delimitation were first reviewed by Siti Nurfazilah et al. (2011) and later expanded by Leong-Škorničková and Boyce (2015). The presence of stolons, indumentum presence and type, shape of staminodial scales, stigma morphology and position, inflorescence/infructescence architecture, colour of the ripe fruit, and morphology and number of seeds per fruit are considered as the most important (Siti Nurfazilah et al. 2011; Leong-Škorničková and Boyce 2015). On the infrageneric level, the most phylogenetically informative traits seem to be the presence of stolons and especially the seed shape (Niissalo et al. 2020). Both so far known stoloniferous species (*H.anthelminthica* and *H.nitens* Siti Nurfazilah, Mohd Fahmi, Sofiman Othman & P.C.Boyce) share shallowly cupuliform seeds and diverged early from the other non-stoloniferous, forest species. The forest species can be further divided into two monophyletic groups, one consisting of robust species with bowl shaped seeds (the so-called large forest species) and the second consisting of small-sized species with almost globose, internally deeply excavated seeds with a wedge-shaped ostiole resembling the popular video game main character called pac-man (the so-called small forest species; Niissalo et al. 2020).

During our field work near Long Tuyo village in Lawas district of Sarawak (northern Borneo) we discovered a small species of *Hanguana* with “pac-man” looking seeds which does not correspond to any described species so far. It is therefore described and illustrated below as *Hanguanadeflexa*.

## Material and methods

This study is based on the material collected in January 2020 in northern Sarawak (Borneo, Malaysia). Morphological characters were studied using hand lens (30–60× magnification) and stereo microscope and documented by macro photography. Collected specimens were thoroughly compared with protologues and types of all *Hanguana* species, with particular focus on species reported from Borneo and species with similar inflorescence and seed morphology. Additionally, herbarium material of *Hanguana* deposited in E, K, KEP, L, P, SAN and SING was studied (herbarium codes according to Thiers 2020). Herbarium vouchers for this study are deposited in SAR and OL. The descriptions follows the style and level of details outlined in Leong-Škorničková and Boyce (2015), while the general terminology is based on Beentje (2016). The preliminary conservation assessments are based on the most recent version of the guidelines of IUCN Standards and Petitions Subcommittee (2019).

We sequenced the tissue of the type collection using ddRADseq with the methods described in full in Niissalo et al. (2020). We mapped the sequences to the consensus sequence published therein, and conducted a phylogenetic reconstruction using 1000 bootstrap replicates with random seed of 1, *Cyrtostachysrenda* as an outgroup, and default settings in RAxML 7.2.8 (Stamatakis 2006). As a test of ploidy levels, we used Smudgeplot v. 0.2.3dev (Ranallo-Benavidez et al. 2020), modified for ddRADseq data as described in Niissalo et al. (2020).

## Taxonomic treatment

### Hanguana deflexa

Taxon classificationPlantaeCommelinalesHanguanaceae

Hroneš & Dančák sp. nov.

0D292574-5EF7-582B-94D8-B963FC8264D2

urn:lsid:ipni.org:names:77219195-1

Figures 1 , 2

#### Diagnosis.

Similar to *Hanguanaloi* Mohd Fahmi, Sofiman Othman & P.C.Boyce but differs in flat leaf blade (vs corrugated), pseudopetioles 0.8–2.1 cm wide (vs to 0.6 cm wide), rachis of infructescence up to 10 cm long (vs ca 58 cm long), ripe fruit ca 1 cm in diam. (vs ca 0.5 cm in diam.), and unbranched partial infructescences (vs branched).

#### Type.

Malaysia, Sarawak, Lawas district, Long Tuyo village, on slopes in primary rainforest above left bank of Tuyo river, ca 2.3 km ESE of village, 4°27.75833'N, 115°29.86000'E, ca 610 m a.s.l., 22 Jan 2020; M. Hroneš & M. Dančák 422020 (holotype SAR, isotype OL).

**Figure 1.**
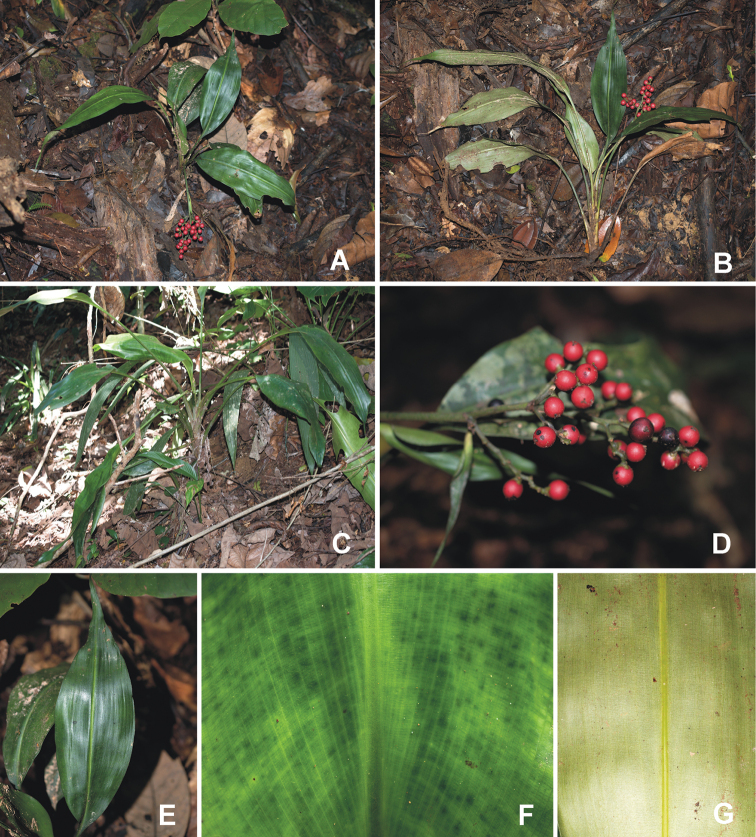
*Hanguanadeflexa* Hroneš & Dančák **A–C** habit in-situ **D** structure of infructescence **E** lamina **F** adaxial and **G** abaxial surface of lamina. Photographed by M. Hroneš (all except **C** from Hroneš & Dančák 422020, **C** from Hroneš & Dančák 452020).

#### Description.

Herbaceous, dioecious mesophyte to ca 1.5 m tall. Stem terete, to 2.5 cm in diam., basally semi-ascending to erect, fully covered by leaf sheaths when young, with age becoming leafless and covered by fibrous remnants of leaf sheaths, terminally with crown of up to 10 leaves; stolons absent. Leaves 70–110 cm long, spreading then arching, bases imbricate with hyaline margins (young leaves), turning brown and erose-marcescent with age; pseudopetiole 13–70 cm long, 0.8–2.1 cm wide, accounting for 1/3–1/2 of entire leaf length, roundly channelled with sharp margins, basally greyish-white floccose; leaf blade 40–70 × 8–20 cm, narrowly elliptic to elliptic, base attenuate, tip long narrowly attenuate with apicule to 5 mm, leathery, flat, adaxially mid to dark green (in close-up with darker green blotches and transverse veins resulting in checker-like variegation), greyish-white floccose when young, falling off in older leaves, abaxially light green, prominently floccose in young leaves, falling off with age; midrib weakly impressed, pale green adaxially, round-raised, lighter green, almost glabrous and shiny abaxially. Male inflorescences not seen, female inflorescence not seen. Infructescence deflexed, procumbent to suberect, comprising of up to 5 partial, whorled, alternate-secund, spiciform infructescences ascending at 70°–80° angle, plus a terminal spike; peduncle and rachis together up to 35 cm tall, pale green when fresh, densely pale brown-grey flocculose, visible portion of peduncle up to 25 cm long; sterile bract one per peduncle, foliaceous, persistent, narrowly ovate to narrowly elliptic with a basal claw, up to ca 14–24 (incl. 2 cm long claw) × ca 3–5 cm; bracts subtending lower partial infructescences similar to sterile bracts, diminishing in size and becoming narrowly triangular distally along the infructescence, the bract supporting the most basal partial infructescence ca 7 × 1 cm (incl. ca 1.5 cm long claw), fully reduced in uppermost partial infructescences; all bracts densely pale brown-grey flocculose; partial infructescences each comprising up to 4 branches at basal levels (gradually less with up to single branch towards the apex of the infructescence), branches arising simultaneously from the axil of the subtending bract, ca 2–3 mm in diam., usually unbranched, up to 5 cm long, with up to ca 10 fruits each. Female flowers scattered, solitary, sessile, all with an associated minute bracteole; perianth composed of 6 tepals in two whorls tightly clasping ovary/fruit in fresh material, all tepals with prominent bulbous thickening at base (more prominent in outer whorl), light green, margin ca 0.5–1 mm wide, hyaline translucent white (turning brown with age); outer tepals broadly ovate, ca 0.5–1 mm long, ca 1.5–2 mm broad, connate at base (only 0.2 mm), sparsely hairy; inner tepals almost orbicular, ca 3–3.5 mm long, 2.5–3 mm broad, basally imbricate but free, sparsely hairy; staminodes 6, in two whorls, pale green, triangular to narrowly triangular, outer staminodes ca 0.5–1 mm long, ca 0.3 mm broad at base, inner staminodes longer, ca 1.5–2 mm long, ca 0.5 mm at base, each basally sheathed with orbicular to broadly ovate, apically pointed to shallowly bilobed scale, ca 1–1.4 mm long, and 0.8–1.2 mm broad, apex and margins semitranslucent (when fresh) to light brown (when dry), gradually darker brown towards the base. Stigma 3-lobed, ca 2.5–3 mm in diam., each lobe ca 1.2–1.5 mm long (fruiting material), ovate to elliptic with slightly pointed apex curved upwards, lobes connate basally with somewhat raised centre, dark brown at fruiting stage, positioned terminally in ripe fruit. Ripe fruit globose, 8–12 mm in diam., dull purplish red, finally turning black; pulp 0.5–2 mm thick, pinkish-white to pinkish-yellow, fairly hard. Seed one per fruit, ca 5–6 × 4 mm, dark brown, ¾ globose to ellipsoid, deeply excavated, ostiole wedge-shaped accounting for ca ¼ of the seeds, without any discernible appendage on the rim, cavity filled with placental tissue.

**Figure 2.**
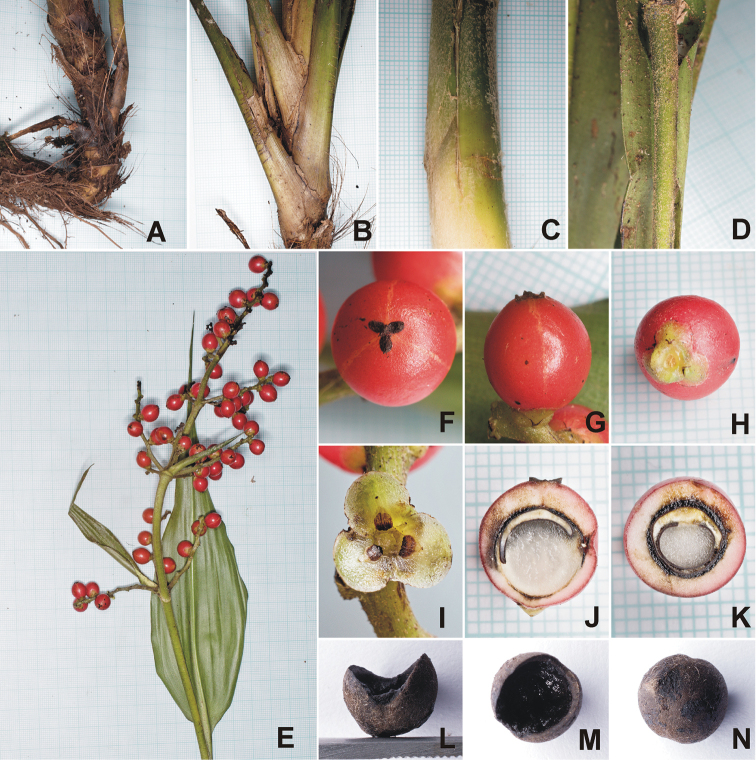
*Hanguanadeflexa* Hroneš & Dančák **A** rhizome with fibre remnants of leaf sheaths **B** stem covered by leaf sheaths **C** floccose indumentum on abaxial side of pseudopetiole **D** peduncle with pale brown-grey floccose indumentum **E** structure of infructescence with sterile and fertile bracts **F** top view of fruit showing detail of stigma **G** side view of fruit **H** basal part of fruit with tepals **I** detail of inner tepals, staminodes and staminodial scales **J** longitudinal section of fruit **K** cross-section of fruit **L** side view of seed **M** top view of seed **N** bottom view of seed. The smallest grid (where applicable): 1×1 mm. Photographed by M. Hroneš (**A, B, D, G–O** from Hroneš & Dančák 422020, **C, E, F** from Hroneš & Dančák 452020).

#### Etymology.

The specific epithet is derived from Latin adjective *deflexus* (i.e. bend, deflexed) and referring to the position of infructescence, a rare character in hitherto described *Hanguana* species.

#### Distribution and habitat.

The species is known only from two localities in primary rainforest southeast of the village of Long Tuyo in Lawas district (northern Sarawak). Both populations occur on moderately steep slopes in altitudes around 600 m a.s.l.

#### Conservation status.

The species is so far known only from two small populations ca 1.4 km apart, each consisting of up to 10 adult individuals. Both populations, considered to be a single location (sensu IUCN Standards and Petitions Subcommittee 2019), are located in the forest with no legal protection and therefore threatened by potential logging. Although it is very likely, that *H.deflexa* occurs in suitable habitats in surrounding areas, it is also clear that the species is not widespread, since no other herbarium collections exist in any of the major herbaria we have examined. Based on the IUCN criteria (IUCN Standards and Petitions Subcommittee 2019), this species shall be provisionally treated as Critically Endangered (CR B2ab(iii); D) until more information is available for easing off this status.

#### Taxonomic remarks.

**Figure 3.**
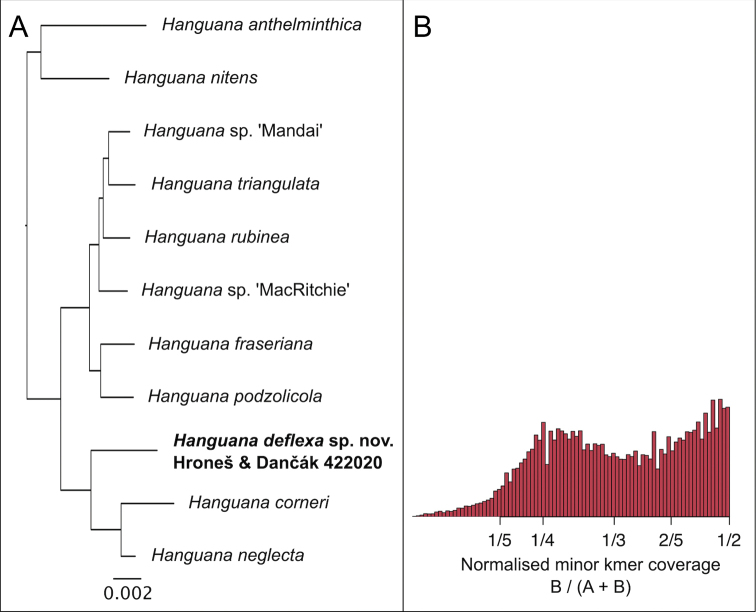
Genomic analyses of *Hanguanadeflexa* Hroneš & Dančák, using ddRADseq **A** maximum likelihood phylogenetic reconstruction using RAxML 7.2.8 (Stamatakis 2006). Sequences were added to a data matrix first published in Niissalo et al. (2020). Outgroups are not shown **B** allele balance, supporting tetraploid status of the species, measured using Smudgeplot v. 0.2.3dev (Ranallo-Benavidez et al. 2020), using kmer length of 21, minimum kmer coverage of 20 and maximum kmer coverage of 10,000.

Based on the seed morphology, *Hanguanadeflexa* belongs to the small forest species clade of *Hanguana* (Niissalo et al. 2020) and therefore it is probably related to *H.corneri* Škorničk. & P.C.Boyce and *H.neglecta* Škorničk. & Niissalo (Niissalo et al. 2014; Leong-Škorničková and Boyce 2015). However, it differs from both species by its deflexed (vs erect) infructescence. It further differs from *H.corneri* by leaf blade abaxially light green (vs purple red) and from *H.neglecta* by fruits ripening purplish red (vs fruits ripening black). The close relationship of *H.deflexa* to *H.neglecta* and *H.corneri* is confirmed in our phylogenetic reconstruction (Fig. 3); however, no other Bornean taxa are yet included in the matrix. The allele balance of *H.deflexa* has a distinctive peak at 25%, suggesting that the species is tetraploid, and, like another tetraploid species, *H.anthelminthica*, it may be capable of sexual reproduction (Niissalo et al. 2020).

The deflexed infructescence is a very rare trait among known *Hanguana* species. Except of *H.bakoensis* Siti Nurfazilah, Sofiman Othman & P.C.Boyce and *H.nana* Randi & Škorničk., all *Hanguana* species have erect infructescences. The protologue illustration of *H.loi* Mohd Fahmi, Sofiman Othman & P.C.Boyce depicts a plant with deflexed infructescence, however, it is described as erect in the main text (Mohd Fahmi et al. 2012). Larger individuals of *H.deflexa* may superficially remind *H.loi* by somewhat arching pseudopetioles and leaf blades (Fig. 1C) and red ripe fruits but both species differ in lamina architecture, width of pseudopetioles, size of ripe fruits and infructescence arrangement as already outlined in the diagnosis. Another similar species, *H.nana*, shares with *H.deflexa* small overall size and red coloured fruits in somewhat sparse, deflexed infructescences but differs by stem evenly covered by leaves and bowl-shaped seeds (Randi et al. 2021).

*Hanguanadeflexa* is already the sixth described species of *Hanguana* known to occur in Sarawak (Mohd Fahmi et al. 2012). Most species are known only from the south-western part of Sarawak, the only exception is *Hanguanamajor* Airy Shaw known from one locality in Lawas district in northern Sarawak. Our field observations suggest that the diversity of *Hanguana* in central and north-eastern parts of Sarawak is much greater and at least ten species occur in this area. However, these species cannot be described at present due to lack of complete photographic evidence and herbarium material of fruiting females necessary for their description.

#### Additional specimen studied (paratype).

Malaysia, Sarawak, Lawas district, Long Tuyo village, on slopes in primary rainforest above right bank of unnamed left-bank tributary of Tuyo river, ca 2 km SE of village, 4°27.37333'N, 115°29.22667'E, ca 620 m a.s.l., 23 Jan 2020; M. Hroneš & M. Dančák 452020 (OL).

### Key to *Hanguana* (Hanguanaceae) in Borneo

Adapted from Mohd Fahmi et al.(2012)

**Table:** 

1a	Colonial helophytes with massive, foliaceous stolons; seeds shallowly cupuliform. Plants of open habitats (banks of large rivers, margins of freshwater bodies)	*** H. anthelminthica ***
1b	Clump-forming mesophytes lacking stolons; seeds bowl-shaped or globose with wedge-shaped ostiole. Plants of closed-canopy forests (lowland to montane rainforests, kerangas)	**2**
2a	Leaf blade corrugated	*** H. loi ***
2b	Leaf blade flat to wavy, not corrugated	**3**
3a	Leaf blade linear-lanceolate to very narrowly elliptic; all parts of the plant (sub)glabrous, without floccose indumentum; fruits weakly 3-lobed, ripening medium pink to magenta	*** H. bakoensis ***
3b	Leaf blade broadly lanceolate, oblanceolate to elliptic, abaxially floccose, at least in juveniles; fruits variable but never 3-lobed, ripening pale yellow, red or black	**4**
4a	Flowering plants small, up to 30 cm tall	**5**
4b	Flowering plants usually 40–150 cm tall	**6**
5a	Stem evenly covered by leaves; lamina elliptic; infructescence deflexed	*** H. nana ***
5b	Stem apically with terminal crown of leaves; lamina broadly lanceolate; infructescence erect	*** H. bogneri ***
6a	Lamina 40–70 cm long; infructescence deflexed; ripe fruits red	*** H. deflexa ***
6b	Lamina 60–100 cm long; infructescence erect; ripe fruits pale yellow or black	**7**
7a	Lamina oblanceolate to narrowly elliptic, with midrib round-raised abaxially; fruit lageniform, ripening black; stigma terminal, prominently stipitate	*** H. major ***
7b	Lamina elliptic, with midrib weakly impressed; fruit globose, ripening pale yellow; stigma distinctly oblique, sessile	*** H. karimatae ***

## Supplementary Material

XML Treatment for Hanguana deflexa
